# Potential for task-sharing to Lady Health Workers for identification and emergency management of pre-eclampsia at community level in Pakistan

**DOI:** 10.1186/s12978-016-0214-0

**Published:** 2016-09-30

**Authors:** Rehana A. Salam, Rahat Najam Qureshi, Sana Sheikh, Asif Raza Khowaja, Diane Sawchuck, Marianne Vidler, Peter von Dadelszen, Shujaat Zaidi, Zulfiqar Bhutta, the CLIP working group, Zia Sultana, Dania Anwer, Payne Beth, Aina Olabisi, Chomiak Marianne, Olukayode A. Dada, Drebit Sharla, Firoz Tabassum, Goudar Shivaprasad, Kariya Chirag, Katageri Geetanjali, Lee Tang, Li Jing, Lui Man Sun, Makanga Tatenda, Ramadurg Umesh, Sharma Sumedha, Solarin Kunle, Laura A. Magee

**Affiliations:** 1 Division of Women & Child Health, The Aga Khan University, Karachi, Pakistan; 2 Department of Research, Vancouver Island Health Authority, Victoria, V8R1J8 Canada; 3 Department of Obstetrics and Gynaecology, and the Child and Family Research Unit, University of British Columbia, Vancouver, V5Z 4H4 Canada; 4 Department of Obstetrics and Gynaecology, St George’s, University of London, London, SW17 0RE UK; 5 Centre for Global Child Health, The Hospital for Sick Children, Toronto, M5G 2L3 Canada; 6 Department of Obstetrics and Gynaecology, Aga Khan University, Stadium Road, Karachi, Pakistan

**Keywords:** Task-sharing, Obstetric care, Community health workers, Community-based interventions

## Abstract

**Background:**

An estimated 276 Pakistani women die for every 100,000 live births; with eclampsia accounting for about 10 % of these deaths. Community health workers contribute to the existing health system in Pakistan under the banner of the Lady Health Worker (LHW) Programme and are responsible to provide a comprehensive package of antenatal services. However, there is a need to increase focus on early identification and prompt diagnosis of pre-eclampsia in community settings, since women with mild pre-eclampsia often present without symptoms. This study aims to explore the potential for task-sharing to LHWs for the community-level management of pre-eclampsia and eclampsia in Pakistan.

**Methods:**

A qualitative exploratory study was undertaken February-July 2012 in two districts, Hyderabad and Matiari, in the southern province of Sindh, Pakistan. Altogether 33 focus group discussions (FGDs) were conducted and the LHW curriculum and training materials were also reviewed. The data was audio-recorded, then transcribed verbatim for thematic analysis using QSR NVivo-version10.

**Results:**

Findings from the review of the LHW curriculum and training program describe that in the existing community delivery system, LHWs are responsible for identification of pregnant women, screening women for danger signs and referrals for antenatal care. They are the first point of contact for women in pregnancy and provide nutritional counselling along with distribution of iron and folic acid supplements. Findings from FGDs suggest that LHWs do not carry a blood pressure device or antihypertensive medications; they refer to the nearest public facility in the event of a pregnancy complication. Currently, they provide tetanus toxoid in pregnancy. The health advice provided by lady health workers is highly valued and accepted by pregnant women and their families. Many Supervisors of LHWs recognized the need for increased training regarding pre-eclampsia and eclampsia, with a focus on identifying women at high risk. The entire budget of the existing lady health worker Programme is provided by the Government of Pakistan, indicating a strong support by policy makers and the government for the tasks undertaken by these providers.

**Conclusion:**

There is a potential for training and task-sharing to LHWs for providing comprehensive antenatal care; specifically for the identification and management of pre-eclampsia in Pakistan. However, the implementation needs to be combined with appropriate training, equipment availability and supervision.

**Trial registration:**

ClinicalTrial.gov, NCT01911494

**Electronic supplementary material:**

The online version of this article (doi:10.1186/s12978-016-0214-0) contains supplementary material, which is available to authorized users.

## Plain language summary

Around ten percent of maternal deaths in Pakistan are attributable to eclampsia. The existing Lady Health Worker (LHW) programme in Pakistan provides an existing platform for early identification and prompt diagnosis of pre-eclampsia in community settings. This study aims to explore the potential of task-sharing to LHWs for the community-level management of pre-eclampsia and eclampsia in Pakistan. A qualitative study was undertaken in two districts of Hyderabad and Matiari involving 33 focus group discussions (FGDs) and review of LHW curriculum and training. LHWs are currently responsible for identification of pregnancies in their community, screening pregnant women for danger signs and referrals. These workers are the first point of contact for women in pregnancy and provide nutritional education along with distribution of iron and folic acid supplements. LHWs do not carry a blood pressure device or medications and refer to the nearest public facility for pregnancy complications. The health advice provided by LHWs is highly valued and accepted by pregnant women and their families. LHW supervisors recognized the need for increased training regarding pre-eclampsia and eclampsia, with a focus on identifying women at high risk. There is a potential for training and task-sharing to LHWs for providing complete antenatal care; specifically for the identification and management of pre-eclampsia in Pakistan.

## Background

Task-sharing involves substituting specialised personnel with healthcare workers who are lesser trained but can perform some aspects of their role [1]. A range of both skilled and semi-skilled health workers can play a major role in maternal, newborn and child health (MNCH) service delivery [33]. Annually around 40 million mothers give birth at home globally without any trained health worker [2, 3]. As a result, most of the maternal, perinatal and neonatal morbidities and mortalities occur at the community level due to lack of good quality care. The poorest countries have the highest maternal and neonatal mortality rates [4, 5]. Within these countries there are dramatic inequalities, with the poorest communities and other marginalised groups experiencing considerably higher rates of maternal and neonatal mortality. The majority of this burden could be averted by achieving universal coverage of good quality care and skilled birth attendance throughout pregnancy and childbirth. However, due to paucity of trained human resource professionals in first-level health services and the reduced awareness of, and accessibility to, services for the deprived and marginalized populations, these are not accessible to those most in need [2, 6]. A more rational distribution of tasks and responsibilities among cadres of health workers is seen as a promising strategy for improving access and cost-effectiveness for MNCH interventions.

Health service delivery through skilled and semi-skilled healthcare workers has been practiced in both high-income countries (HICs) and low- middle- income countries (LMICs) for several decades. More recently, due to the growing human resource crisis especially in LMICs, task-sharing has re-emerged for extending services to hard-to-reach groups [7–12]. Countries in South Asia and Africa have made a particular effort in recent years to reduce maternal and neonatal mortality and morbidity through deploying community health workers (CHW) [13–16]. The role of midwives and traditional birth attendants (TBA) in delivering MNCH services has also received growing attention, and a number of publications have described their role and documented the effects of such programmes [17, 18, 32]. Furthermore, utilising these alternate cadres of healthcare workers is widely recognised as an important strategy to deliver key MNCH interventions in many LMICs [19–21]. CHWs can be used to deliver a wide spectrum of promotional and preventive interventions including provision of basic antenatal care (ANC), essential newborn care, breastfeeding counselling, management and referral of sick newborns, and community mobilisation. It also provides a channel to reach far flung areas, thus attempting to reduce existing inequities in healthcare access and utilization.

Pakistan has not meet the Millennium Development Goals (MDGs) for maternal and child health by 2015. An estimated 276 Pakistani women die for every 100,000 live births, and 48 % of deliveries occur without assistance from a skilled care provider [22]. Pre-eclampsia and eclampsia account for about 10 % of the overall maternal mortality in Pakistan. CHWs are a part of the existing health system in Pakistan in the form of the Lady Health Workers (LHWs). The LHW Programme was introduced in the public service infrastructure in Pakistan in 1994 and currently employs 96,000 LHWs. Under this banner, public health sector employs LHWs and Lady Health Visitors (LHVs) in rural sectors with 15 months of training in MNCH. Each of these LHWs covers a population of 1000–1500 [23, 24]. These LHWs are usually women residing in the surrounding locality. In the existing health system, the role of LHW is to provide a comprehensive package of antenatal care including pregnancy identification, basic health education, identification of the danger signs of pregnancy and referral when needed; and provide minimal neonatal care. However, there is a need of increased focus on early identification and prompt diagnosis of pre-eclampsia in community settings, since women with non-severe pre-eclampsia typically present with no visible symptoms. This leads to delays in diagnosis, adequate primary care, and referral; consequently contributing to adverse maternal and fetal outcomes.

The Community Level Intervention for Pre-eclampsia (CLIP) trial package will train the existing LHWs for community mobilisation; identification and triage of women with pre-eclampsia (through weight, blood pressure and urine for proteinuria); administration of oral antihypertensive agent when indicated (methyldopa); administration of intramuscular MgSO4 when indicated and timely referral to facility for women within their catchment population. The objective of this study is to explore the potential for task-sharing to community healthcare providers and LHWs, for early identification and management of pre-eclampsia and eclampsia and implementation of CLIP package in Pakistan.

## Methods

A qualitative exploratory study was undertaken between February-July 2012, as part of a large multi-country study - Community Level Intervention for Pre-eclampsia (CLIP) trial (NCT01911494). The study was conducted in two districts, Hyderabad and Matiari, in the southern province of Sindh, Pakistan. Hyderabad is an urban district located and the second largest city in Sindh province with a population of over 3 million. The rural district Matiari is located 25 km north of Hyderabad [25]. The total population of Matiari exceeds half a million, with large rural settlements [26]. The study participants included LHWs, Lady Health Supervisors (LHSs), women medical officers (WMO), and TBAs. Altogether 33 focus group discussions (FGDs) were conducted: seven with LHWs, ten with LHSs, nine with WMOs, and seven with TBAs. The LHW curriculum and training materials were also reviewed. Participation in this study was voluntary, and written consent was obtained from all participants prior to data collection.

The data were collected through the FGDs as it allows participants in a small group to discuss the subject freely and spontaneously [27, 28]. FGD guides were developed for a priori themes informed by the literature; all questions were translated into Sindhi and Urdu languages. These questions were pilot tested for comprehension, cultural sensitivity, appropriateness and duration. All the FGDs were moderated by research officers, accompanied by two research assistants for note-taking. The project staff were native Sindhi or Urdu speakers, they were locally recruited and trained by a senior faculty and a social scientist with qualitative research expertise. The FGDs were audio-recorded; field notes were also taken. Discussions were transcribed verbatim into Sindhi or Urdu. Stringent data quality control measures were followed, which included random observations of FGDs by field coordinator; audit-trailing of 20 % of the FGDs to verify audio with typed transcripts; and biweekly debriefing sessions with moderators and transcribers. In addition, the moderator maintained a self-reflection after each session to describe their personal opinions to contextualise the data, as well as, preventing the self-bias.

A self-administered questionnaire was completed by 457 LHWs, all questions were designed using a Likert scale (strongly disagree to strongly agree). For reporting purpose, the responses ‘strongly agree’ and ‘agree’ were merged together and reported here as an affirmative response to the question. A response was considered to be negative if answers were either ‘strongly disagree’ or ‘disagree’. The competency and skills of LHWs was evaluated by a set of questions pertaining to their current responsibilities: ability to identify danger signs of pregnancy, blood pressure (BP) measurement, administration of injections at home, and acceptance of referral in community.

Administrative and logistic support for task-sharing was evaluated by review of LHW training.

Using NVivo 10 [QSR, Doncaster Vic, Australia], all participants’ responses were coded to relevant themes. Subsequently, emerging themes and sub-themes were drawn from the initial structure.

This study received ethical approval from Ethics Review Committee of Aga Khan University, Karachi Pakistan (1917-Obs-ERC-11) and Institutional Review Board of University of British Columbia, Vancouver Canada (H12-00132).

## Results

### Maternal health responsibilities

A review of the LHW curriculum and training highlight that contact with pregnant women for the purpose of health education is a part of their current job description. LHWs are local residents, responsible for registration and follow-up of pregnant women and new-borns; they are the first contact for health care. Their main responsibility in pregnancy is to counsel women regarding a healthy diet and antenatal care; to inform of danger signs of pregnancy; highlight the importance of exclusive breastfeeding; and provide care for the newborn. LHWs are expected to refer pregnant women to the local health facility if they identify any danger signs. In addition, health advice, the role of LHWs is to assist families to plan for the expenditures associated with pregnancy and delivery. LHWs conduct regular education and awareness sessions in community on topics related to existing health problems. Currently they do not prescribe any medications apart from tetanus toxoid immunization for pregnant women and vaccinations for newborns and children (Table 1).

**Table 1:** Lady Health Worker National Programme for family planning and basic health

Lady Health Worker Maternal Health Responsibilities
• Counsel and vaccinate all women (15–49 years of age) of their area for tetanus
• Keep record of pregnant women of their respective area for advice and referral
• Advise pregnant women about: o Healthy diet o Accessing antenatal care from a health facility o Exclusive breast feeding o Uptake of offered iron and folic acid tablets
• Maintain liaison with TBAs in their area for safe birth and to refer women to local health facility as required
• Identify danger signs during pregnancy, delivery and postpartum and refer appropriate women to a health facility

During FGDs, LHWs discussed some of their job responsibilities as outlined in the curriculum: identification of pregnancies, registration of pregnancies, periodic visits to encourage birth preparedness, dietary advice and provision of multi-vitamin tablets. LHWs do not carry any antihypertensive agents for pregnant women; in cases of suspected or confirmed hypertension they refer to the nearest public facility. LHWs report regularly to LHSs, who confirm their registers. In FGDs with LHWs, one participant said “*If she has high blood pressure, bleeding and water comes out, and then refer her to the hospital*”. In another FGD with LHWs, one participant narrated that “*If we see dangerous signs in pregnant women then we informed her that these are the signs: tension, she does lots of vomits and because of that BP becomes high, because of vomits; fits can occur. On that time we recommend them to go to health facility*”. Referral was also endorsed by LHS on interview that “*We usually don’t handle these types of cases as we don’t have the equipment to cater to the problem properly. We usually refer them to a lady hospital, CDF or Bhattai Hospital*”.

### Competence in providing maternal health services

Findings from LHW-administered questionnaires suggest that they are able to identify danger signs in pregnancy and refer women (Table 2). During FGDs, LHWs stated that they register pregnant women and make episodic home visits; therefore, over time they develop a good rapport with the pregnant women and their mother-in-laws. The large majority (94 %) of LHWs reported that families accept their health advice or referrals. Although acceptance of referral was reported by 432 (94 %) of LHW, only 44 (10 %) believed that pregnant women would accept an injection from them before referral in cases of pre-eclampsia or eclampsia. The majority of the LHWs (82 %) reported that they can refer pregnant women directly to a district level hospital. LHWs expressed confidence in basic skills in providing maternal care: 34 % were confident in measuring BP and 87 % were confident in administering intramuscular injections. Around 44 % of the LHWs mentioned receiving training to identify pregnancy complications while 56 % mentioned receiving training to refer or manage pregnancy complication (Table 2).

**Table 2:** Lady Health Worker competencies and skills related to maternal health care

Competency/Skills	Frequency (%) of affirmative responses *n* = 457
Identify pregnancy signs and symptoms	457 (100 %)
Identify danger signs of hypertension in pregnancy	331 (72 %)
Identify danger signs of seizures in pregnancy	422 (92 %)
Can measure BP	153 (34 %)
Can administer intramuscular injections	389 (87 %)
Can refer pregnant women to District Hospital	376 (82 %)
Acceptance by pregnant women or decision-making proxies of referral	432 (94 %)

Despite the confidence expressed, only 9 % of LHWs claimed to have a BP apparatus; only 4 % had access to pregnancy tests; 24 % had a measuring tape and 42 % had weighing scales (Table 3).

**Table 3:** Administrative and logistic support available to Lady Health Workers to provide maternal health care

Administrative support	Frequency (%) of affirmative responses *n* = 457
Trainings to identify pregnancy complications	203 (44 %)
Trainings to refer or manage pregnancy complication	254 (56 %)
Available tools for screening of pre-eclampsia	
BP apparatus	38 (9 %)
Weight machine	183 (42 %)
Urine dipstick (pregnancy test)	22 (4 %)
Measuring tape	108 (24 %)

### Gaps in training

Findings from questionnaires and FGDs suggest a need for periodic training regarding patient triage and the emergency management of pre-eclampsia and eclampsia. Many LHSs recognised the need for training, with a focus on identifying woman at high-risk. Also, they claimed that pre-eclampsia and eclampsia are very important subjects; however, they had received negligible attention during their training. In contrast, very few TBAs perceived the need to learn about pre-eclampsia and eclampsia.

### Government and facility healthcare provider support

Facility providers offer backup for LHW referrals. They do not make regular home visits but they support LHWs in the community. The LHW Programme, outlined by the Government of Pakistan, indicates strong support by policy makers for community care provision by LHWs. During an IDI with women medical officer, she narrated that “*We motivate them to get their check-up done regularly. If they are hypertensive then we give them medicine weekly and tell them to get their weekly check-up done*”.

## Discussion

Findings from this study demonstrate that the existing LHW program could provide a platform for community-based identification and management of pre-eclampsia and eclampsia in Pakistan with the help of the existing network of LHWs. LHW have the capacity to screen women for pre-eclampsia, administer emergency medications and refer with appropriate training. The potential task-sharing to community level delivery of these services is reasonable as LHWs already perform regular home visits and deliver health promotion, preventive care and essential curative MNCH services. LHWs conduct visits during pregnancy and encourage families and communities to gain timely access to referral facilities. There is significant community acceptability of LHWs’ capacity to deliver additional services. There was insufficient LHW training related to the prevention and management of pre-eclampsia and eclampsia specifically.

The World Health Organization and the International Federation of Gynaecology and Obstetrics (FIGO) recognize the critical role community and lay health workers play in preventing post-partum haemorrhage and increasing access to misoprostol where skilled birth attendants are not available [34]. Existing literature supports the findings of our feasibility study and suggest that task-sharing to community health workers for antenatal care delivery has shown some evidence of success [31]. In Pakistan, antenatal care delivery through LHW and TBA in community settings has shown improvement in maternal morbidity and mortality [35, 36]. A large scale community-based study in Pakistan on LHW training to provide antenatal care alongside TBA showed favourable results [37]. Another study on educating and training mothers through LHW resulted in a greater number of visits from the LHW in the intervention group as compared with the routine antenatal care package delivery group [37]. There are successful examples of community based trails where community health workers have been trained to administer medications. A study involving TBA, LHW and other community workers to administer 600 micrograms of misoprostol in women with postpartum haemorrhage has shown an impressive 24 % decrease in maternal mortality [38]. This is supportive of the recommendation that with proper training, it is feasible to incorporate emergency medication administration into the LHW care package, in community settings where accessibility and availability are an issue. There is also precedence of task-sharing where LHWs have provided misoprostol to reduce vaginal bleeding and administered oral amoxicillin to children under 5 for pneumonia at home [39]. These findings highlight that administration of methyldopa and MgSO_4_ by LHWs is feasible as planned for the CLIP study. In addition, this approach is supported by the World Health Organization (WHO) in terms of providing technical assistance, including the development of manuals, training activities, improved supervision and monitoring, resource generation and capacity building of LHW [29].

LHWs play a pivotal role in Pakistan’s health care system [30, 40]; however the existing number of LHW is estimated to be insufficient according to the World Health Organization [29]. This human resource shortage must be considered in the redistribution of health care services. Furthermore, with the annual income of around $343, LHWs often struggle to satisfy the financial needs of their families, which is made more difficult as more responsibilities are added to their role [29, 30]. There is a need to critically review the current tasks assigned to LHWs prior to the inclusion of additional activities. If LHWs are to identify pre-eclampsia, they must be provided a blood pressure apparatus and urine dipsticks for assessment and diagnosis in community settings. In addition, it is important to create acceptance within the community for emergency treatment to be given by LHWs. In order to strengthen the delivery of these new interventions the focus should be on skills development and supportive supervision. Skills development and competencies need to be enhanced for blood pressure monitoring, dipstick testing of urine, and medication administration. Task-sharing of this nature, will require high levels of motivation, focus and accountability on the part of LHWs. Additional motivation and reinforcement are needed to use protocols where they exist for managing patients with pre-eclampsia, and novel education strategies are required where such protocols do not exist.

## Conclusion

There is strong potential for training and task-sharing to LHWs for providing comprehensive antenatal care; including the identification and management of pre-eclampsia in Pakistan. However, implementation must be coupled with appropriate training, equipment provision and supervision.

## Additional file

Additional file 1: Reviewer reports. (PDF 158 kb)

## Abbreviations

ANC: Antenatal care
BP: Blood pressure
CHW: Community health workers
CLIP: Community Level Intervention for Pre-Eclampsia
FGD: Focus group discussions
FIGO: International Federation of Gynaecology and Obstetrics
HIC: High-income countries
LHS: Lady Health Supervisors
LHV: Lady Health Visitors
LHW: Lady Health Workers
LMIC: Low- middle- income countries
MDG: Millennium Development Goals
MNCH: Maternal Newborn and Child Health
TBA: Traditional Birth Attendants
WHO: World Health Organization
WMO: Women medical officers
