# Supplementary material for: Potential for task-sharing to Lady Health Workers for identification and emergency management of pre-eclampsia at community level in Pakistan
Source: Reprod Health. 2016 Sep 30;13(Suppl 2):107. doi: 10.1186/s12978-016-0214-0 (PMC5056493; doi:10.1186/s12978-016-0214-0)
Supplement: Additional file 1: — Reviewer reports. (PDF 158 kb) [file 12978_2016_214_MOESM1_ESM.pdf]

Referee's comments to the authors– this sheet WILL be seen by the author(s) and published with the article

Title	Potential for task-shifting to Lady Health Workers for identification and emergency management of pre-eclampsia at community level in Pakistan
Author(s)	Rehana A Salam, Rahat Najam Qureshi, Sana Sheikh, Asif Raza Khowaja, Diane Sawchuck, Marianne Vidler, Peter von Dadelszen, Shujaat Zaidi, Zulfiqar Bhutta and the CLIP working group
Referee's name	Claudia Diaz

When assessing the work, please consider the following points, where applicable:

1. Is the question posed by the authors new and well defined?
2. Are the methods appropriate and well described, and are sufficient details provided to replicate the work?
3. Are the data sound and well controlled?
4. Does the manuscript adhere to the relevant standards for reporting and data deposition?
5. Are the discussion and conclusions well balanced and adequately supported by the data?
6. Do the title and abstract accurately convey what has been found?
7. Is the writing acceptable?

Please make your report as constructive and detailed as possible in your comments so that authors have the opportunity to overcome any serious deficiencies that you find and please also divide your comments into the following categories:

- Major Compulsory Revisions (which the author must respond to before a decision on publication can be reached)
- Minor Essential Revisions (such as missing labels on figures, or the wrong use of a term, which the author can be trusted to correct)
- Discretionary Revisions (which are recommendations for improvement but which the author can choose to ignore)

Where possible please supply references to substantiate your comments.

When referring to the manuscript please provide specific page and paragraph citations where appropriate.

General comments: The authors have selected a major cause of MM in lower and middle income countries; hypertensive disorders of pregnancy with an emphasis on pre-eclampsia/eclampsia. They have documented what similar studies in similar settings have found, mainly that task-shifting is a feasible, needed and cost-effective way of reducing MM and increase the cadre of trained health personnel. They also point out the deficiencies in a system that underpays and over burden health care personnel and often does not equip them with the most basic tools to carry our early identification of warning signs or the medication needed to stop symptom progression.

Major compulsory revisions:

Minor essential revisions: If available, it would be interesting if the guidelines for service provision that according to the text were introduced in Pakistan in 1994 have undergone revisions.

Discretionary revisions: If the data from the multi-country study include HELLP syndrome and near miss cases, it would be interesting to mention if LHW participate in their management of if these are only referred and managed at the hospital level. If the data is not available it is understandable they are not the focus of this study

(continue on the next sheet)

Referee's comments to the authors– this sheet WILL be seen by the author(s) and published with the article

Title	Potential for task-shifting to Lady Health Workers for identification and emergency management of pre-eclampsia at community level in Pakistan
Author(s)	Rehana A Salam, Rahat Najam Qureshi, Sana Sheikh, Asif Raza Khowaja, Diane Sawchuck, Marianne Vidler, Peter von Dadelszen, Shujaat Zaidi, Zulfiqar Bhutta and the CLIP working group
Referee's name	Salis Mohammed Ishaku

When assessing the work, please consider the following points, where applicable:

1. Is the question posed by the authors new and well defined?
2. Are the methods appropriate and well described, and are sufficient details provided to replicate the work?
3. Are the data sound and well controlled?
4. Does the manuscript adhere to the relevant standards for reporting and data deposition?
5. Are the discussion and conclusions well balanced and adequately supported by the data?
6. Do the title and abstract accurately convey what has been found?
7. Is the writing acceptable?

Please make your report as constructive and detailed as possible in your comments so that authors have the opportunity to overcome any serious deficiencies that you find and please also divide your comments into the following categories:

- Major Compulsory Revisions (which the author must respond to before a decision on publication can be reached)
- Minor Essential Revisions (such as missing labels on figures, or the wrong use of a term, which the author can be trusted to correct)
- Discretionary Revisions (which are recommendations for improvement but which the author can choose to ignore)

Where possible please supply references to substantiate your comments.

When referring to the manuscript please provide specific page and paragraph citations where appropriate.

General comments:

This paper addresses an important cause of maternal and newborn mortality and morbidity not only in Pakistan but globally. The Lady Health Workers program is an example of community-based platforms that should be utilized to reach families in hard-to-reach locations with live-saving health information and services. This paper should be published in order to reach a wider audience of health programmers and policy-makers. However, the manuscript would benefit from further revision for language and proper placement of punctuation marks.

Major compulsory revisions:

Discussion

Line 216 – 218:

"Findings from this study demonstrate that community-based identification and management of pre- eclampsia and eclampsia is feasible in Pakistan with the help of the existing network of LHWs. LHW have the capacity to screen women for pre-eclampsia, administer emergency medications and refer"

There is nothing in this study results that showed that LHW have the capacity to screen women for pre-eclampsia and administer emergency medication. Although 94% of the LHW interviewed believed they could administer intramuscular injection, they haven't done that yet. In fact to contradict the paragraph above, line 223 said that "There was insufficient LHW training related to the prevention and management of pre-eclampsia and eclampsia specifically" So, this segment should be revised.

Minor essential revisions:

Background line 32 – 33, I would recommend a change from “since women with moderate pre-eclampsia often present without symptoms” to since women with mild pre-eclampsia often present without symptoms”. The current classification of pre-eclampsia does not include moderate pre-eclampsia. It is only mild and severe pre-eclampsia.

Line 94: I will also recommend a change from “Pakistan will not meet the Millennium Development goal for women and children by 2015” to “Pakistan has not met the ………………………. By 2015”. The 2015 MDG timeline is now in the past.

Line 110 – 117: The three sentences within these lines are not very clear to me. The first paragraph indicated that the CLIP utilizes the LHWs platform for community mobilization; identification and triage of women with pre-eclampsia; administration of oral antihypertensive agent when indicated (methyldopa); administration of intramuscular MgSO4 when indicated and timely referral to facility. This indicates that the LHW are already being trained to do the job. Then the next sentence said t they can be trained to assess weight, blood pressure and proteinuria…….. This sounds confusing or contradictory. Finally, the sentence that starts in line 115 said “ the objective of this study was to explore the feasibility of ………..”. I think feasibility is not the right word to use as it means capability which this study did not show. It would be nice if the authors stay with the wording used in the title of the manuscript. That is to say that the “objective of this study is to explore the **potential of**…”
